# Resistive Switching Memory Phenomena in PEDOT PSS: Coexistence of Switchable Diode Effect and Write Once Read Many Memory

**DOI:** 10.1038/srep19594

**Published:** 2016-01-25

**Authors:** Viet Cuong Nguyen, Pooi See Lee

**Affiliations:** 1 School of Materials Science and Engineering, Nanyang Technological University, 50 Nanyang Avenue, Singapore 639798, Singapore

## Abstract

We study resistive switching memory phenomena in conducting polymer PEDOT PSS. In the same film, there are two types of memory behavior coexisting; namely, the switchable diode effect and write once read many memory. This is the first report on switchable diode phenomenon based on conducting organic materials. The effect was explained as charge trapping of PEDOT PSS film and movement of proton. The same PEDOT PSS device also exhibits write once read many memory (WORM) phenomenon which arises due to redox reaction that reduces PEDOT PSS and renders it non-conducting. The revelation of these two types of memory phenomena in PEDOT PSS highlights the remarkable versatility of this conducting conjugated polymer.

Organic materials have been investigated intensely for memory applications due to their low cost, flexibility and versatility. Coupling with the two terminals resistive memory architecture, the organic resistive memory device offers simple structure and flexibility for applications such as RFID tag and data archives^1^. Various works have demonstrated interesting resistive memory phenomena in polymer and composites such as Dynamic Random access memory (DRAM)^2^, Static Random Access Memory (SRAM)^3^, Write Once Read Many Memory (WORM)^4^ and Rewritable memory (FLASH)^5^. The mechanisms behind these memory phenomena are often complex and depend strongly on the top electrode materials or deposition condition; the mechanisms are normally suggested by first principle calculations^6^. There are various mechanisms suggested for resistive switching phenomena in organic materials namely, donor-acceptor charge transfer complex^3^, charge trapping due to redox reaction^7^ and modulation of dopant in conjugated polymer^8^ or electrode metal migration^9^,^10^.

In this work, we study resistive switching phenomena in conducting polymer poly (3,4-ethylene-dioxythiophene):poly(styrenesulfonate) (PEDOT:PSS) where poly styrenesulfonate acid PSS H provides doping for conjugated polymer poly (3,4-ethylene-dioxythiophene) PEDOT and makes it water dispersible. The WORM memory characteristic of PEDOT : PSS can be attributed to the modulation of dopant PSS H and redox reaction^11^. Recently, we have also shown that PSS H doped polyaniline (PANI) also demonstrated WORM memory characteristic^12^. Alongside with WORM memory, PEDOT PSS has been investigated for applications such as rewritable memory/synapsis activities^13^,^14^,^15^. In most cases^13^,^14^,^15^,^16^,^17^, the role of PEDOT PSS was minor while memory function was driven by active electrodes such as Ag or metal oxide of Ti, Ta or Al. In this work, we show that switchable diode effect can be observed in micrometer thick PEDOT PSS film through charge trapping and cation movement characteristics in the thick PEDOT PSS; these activities were originated from PSS phase separated regions within the PEDOT PSS film. Fundamentally, this switchable diode effect is very different from those observed in ferroelectric diode, or metal oxide such as WO_3_, TiO_2_ and SnO_2_^18^,^19^,^20^,^21^. Furthermore, WORM memory phenomenon was also observed in the same device configuration due to the redox reactions that reduces PEDOT^+^ to PEDOT^0^.

## Results and Discussion

### Original resistance state and Write Once Read Many Memory

**Figure 1:**
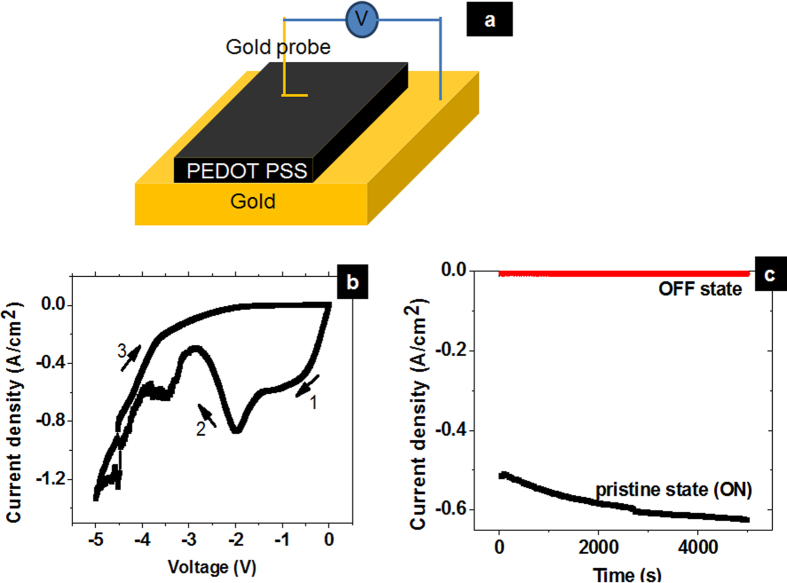
(**a**) Experimental set up configuration. (**b**) Continuous voltage sweep from 0 V to −5 V of pristine PEDOT PSS film (**c**) long retention characteristic of the WORM device.

The current voltage (I-V) characteristic collected using metal probe as top electrode in the Au-PEDOT PSS-Au configuration was shown in Fig. 1a. Originally, the film was in its high conducting state with Ohmic behavior. Figure 1b shows the I-V sweep from 0 V to −5 V which exhibits a hysteresis where current transits from high conducting state to low conducting state which is similar with I-V hysteresis of PANI PSS^12^ and PEDOT PSS observed by Bhansali *et al.*^22^. After this sweep, read voltage at lower bias of 0.5 V shows a low conducting state. The device was unable to resume to the conducting pristine state by applying higher negative or positive bias. The result indicates a write once read many memory phenomenon where the pristine state can be written, but the written state cannot be erased. The long retention of the write once read many memory is shown in Fig. 1c where conducting state of the pristine film (ON state) and film after applying −5 V (OFF state) can be maintained for more than 5000 s with no degradation; the states were read at −0.5 V. The retention was monitored again after 3 months and shows no change as shown in supplementary information Fig. S1. The write once read many memory mechanism in PEDOT PSS has been attributed to redox reactions that reduce PEDOT^+^ to PEDOT^o^ in the bulk film^11^. We recorded optical image of the pristine PEDOT PSS film and after biasing −5 V as shown in Fig. S2a. The region biased by −5 V showed a darker color compared with pristine region which implies the occurance of electrochemical reduction. It is noted that the dark region is not due to mechanical means such as probing process but due to electrical biasing at −5 V. We did not observe the black region after biasing 5 V. Raman spectroscopy is a convenient and powerful tool to investigate electronic properties of conducting polymer after biasing. Intensities of Raman peak at 1267 cm^−1^ and band shape near 1450 cm^−1^ can be useful indicator of doping and dedoping of PEDOT PSS^23^. Subsequent investigation of the dark area by Raman spectroscopy showed the disappearance of Raman peak at 1267 cm^−1^ and shoulder peak at 1400 cm^−1^ (Fig. S2b) after biasing −5 V; this fact further indicates that the biased area has been reduced^23^,^24^. Furthermore, as shown in Fig. S3, current density peak at −2 V in Fig. 1b is sensitive to the rate of voltage sweep. As the sweep speed is increased, the current density level at −2 V will increase which suggests that the electrochemical reactions play an important role.

### Switchable diode memory effect

**Figure 2:**
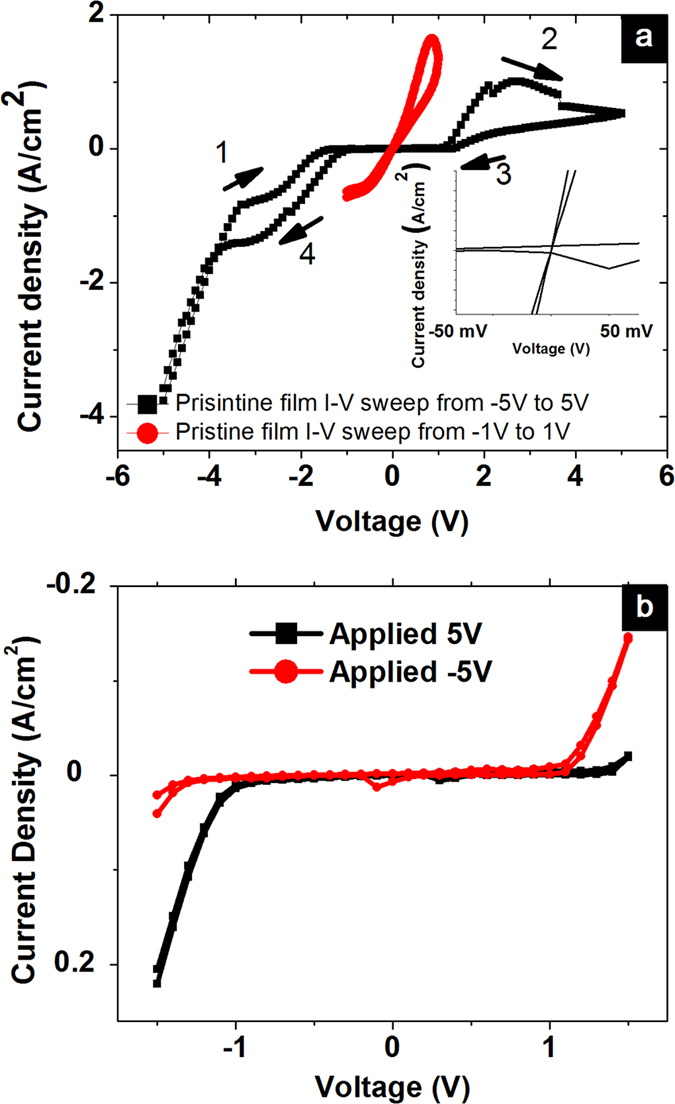
(**a**) Continuous voltage sweep from −1 V to 1 V and −5 V to 5 V applying on pristine film independently (**b**) Voltage sweep at low bias (−1.5 V to 1.5 V) after applying voltage on the device by 5 V or −5 V.

The switchable diode effect arose only after negative voltage sweep in Fig. 1b where original high conducting state (pristine state) at low bias (−1 V or −0.5 V) was suppressed irreversibly. To correlate these two effects, we applied voltage sweep from −1 V to 1 V and from −5 V to 5 V to the pristine film independently. We show in Fig. 2a, the I-V characteristic of the pristine film after voltage sweeping from −1 V to 1 V and voltage sweeping from −5 V to 5 V. As seen from Fig. 2a, the I-V characteristic has Ohmic conduction at low bias (0.5 V) when voltage was swept from −1 V to 1 V; however sweeping from −5 V to 5 V indicates bistable hysteresis and almost zero current at low bias (0.5 V). Zooming in very low voltage regime (−50 mV to 50 mV), we observe that for voltage sweep from −1 V to 1 V, the I-V curve exhibits Ohmic properties with the I-V curve crosses axis origin while after voltage sweep from −5 to 5 V, the I-V curve does not cross axis origin as shown in inset of Fig. 2a. As seen from Fig. 2a, the switchable diode effect arises after biasing from −5 V to 5 V. Continuous voltage sweep from −5 to 5 V indicates two current peaks at positive and negative bias regime which are the characteristics of negative differential resistance (NDR). The NDR is often observed in trap/detrap resistive switching device^25^ or molecular junction with redox centers^26^. For better representation, we measured I-V at low voltage (from −1.5 V to 1.5 V) after writing with 0.1 s duration rectangular pulse of −5 V and 5 V; the switchable diode effect was observed as shown in Fig. 2b. After applying voltage to the device by 5 V or −5 V, reversing of rectification can be seen by small reading voltage sweep from −1.5 V to 1.5 V with rectification ratio of 10 and 3.5 for pulse of 5 V and −5 V, respectively.

Unlike the similar phenomenon in ferroelectric diode^18^ where no NDR was observed, in this case the switchable diode effect is accompanied by two NDR regions. Furthermore, in Fig. 2b, the diode forward polarity is opposite to the writing pulse polarity which is in contrast with the similar phenomenon in ferroelectric diode where the diode forward direction follows the writing pulse polarity^18^. It is the first time switchable diode effect was observed in conducting polymer system such as PEDOT PSS.

**Figure 3:**
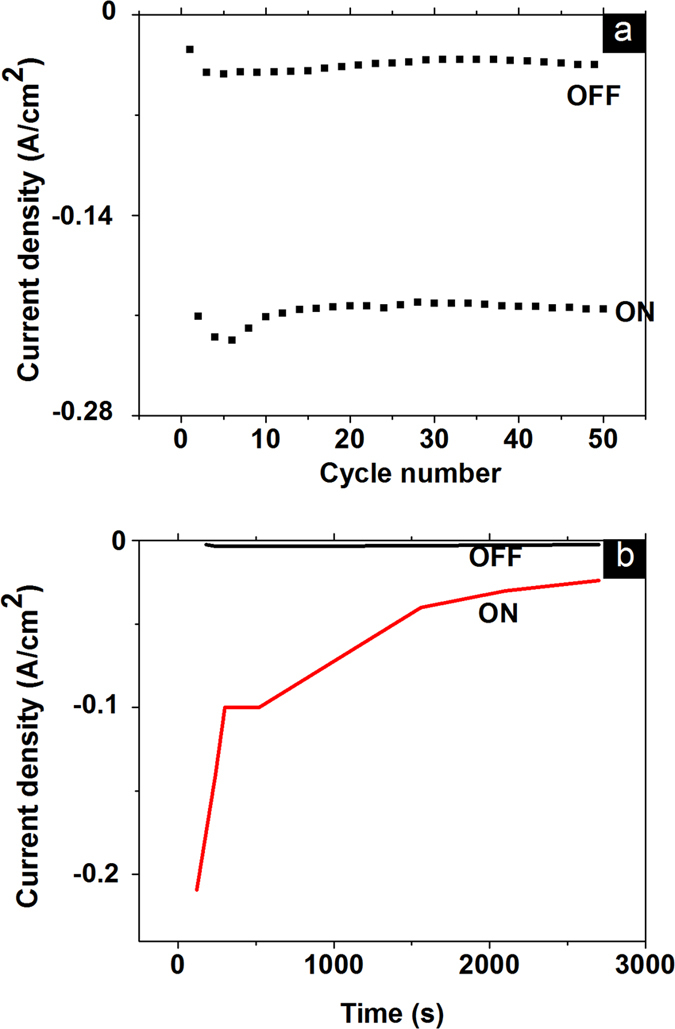
Cycling and retention characteristics of Au-PEDOT PSS-Au junction. (**a**) ON-OFF state cycling (**b**) retention characteristics of the Junction.

The states retention and write/erase cycles were shown in Fig. 3. We recorded ON/OFF ratio and states retention at −1.7 V after writing 5 V or −5 V respectively for 60 seconds. ON and OFF state can be switched for more than 50 times and resistive states can be maintained for more than 3000 seconds. The data lost in Fig. 3b replicates the short term memory discussed largely in memristive systems such as WO_3_ and SnO_2_^19^,^21^. The short term memory fits to category of organic static random access memory discussed in ref. 3. In static random access memory, memory states can be retained for hours and eventually lost if device is not powered. Similar trend of state retention was also observed in switchable diode of WO_3_ and SnO_2_^19^,^21^. The results are highly repeatable with 100% device yield for 20 tested junctions from different batches. The above retention and cycling results indicate nonvolatility of this memory phenomenon. In Fig. S4, distribution of On state current and Off state current over 20 tested devices from different batches is shown. The On state and Off state current can be clearly distinguished.

**Figure 4:**
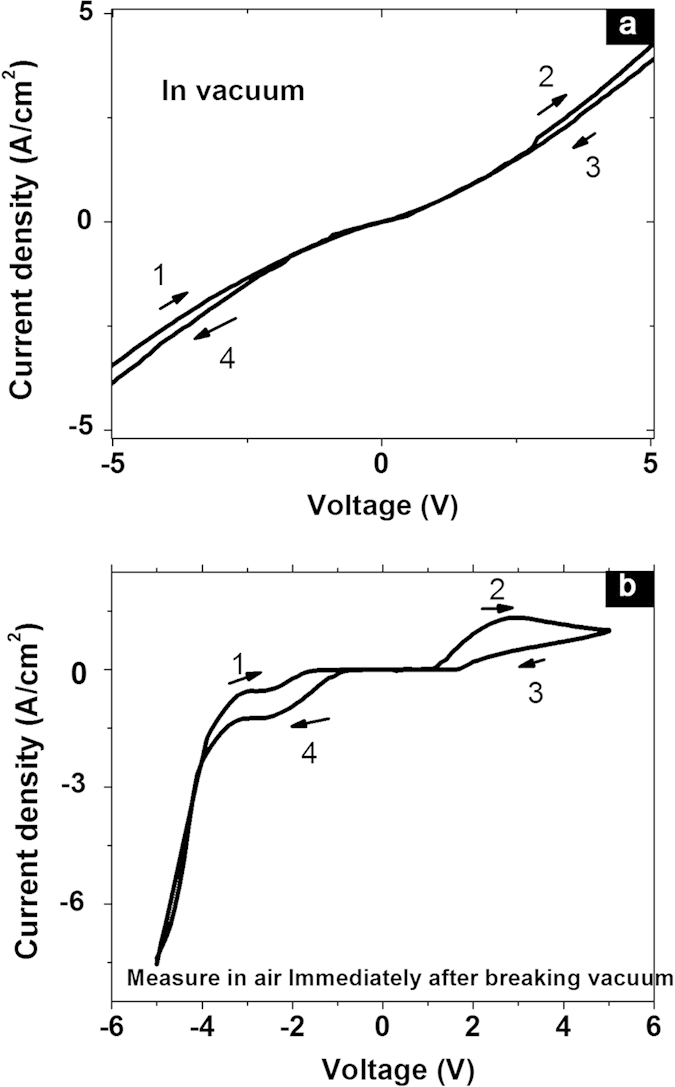
(**a**) I-V measurement at 10^−4^ torr (**b**) I-V measurement in air immediately after taking out the sample from vacuum.

To understand the working mechanism of this switchable diode memory phenomenon, the junction was further tested in vacuum at 10^−4^  torr; the I-V shape as shown in Fig. 4 is almost a straight line when voltage was swept from −5 to 5 V. Compared with device tested in air, the lack of I-V nonlinearity and hysteresis suggests that water molecules or oxygen traps in the film may have contributed to the observed switchable diode and bistable switching. After testing in vacuum, the device was tested in air again; the voltage was swept from −5 to 5 V and the I-V shape returned completely to its original form as shown in Fig. 2a. Therefore, we deduce that the adsorbed water molecules are possibly the main reason for switchable diode effect in Fig. 2a,b. Observation of I-V hysteresis in some hygroscopic insulator field effect transistor device in humid air was reported and the phenomenon was extinct when device was tested in N_2_ gas^27^. Similarly, adsorbed water molecules induced memory phenomenon was also reported in carbon nanotube field effect devices^28^; in this scenario, I-V hysteresis was observed when device was tested in air and extinct when it was done in vacuum.

We performed control experiments to validate this phenomenon. I-V response of Poly(4-styrenesulfonic acid) (PSSH) was recorded. In Fig. S4a, the I-V sweep from −5 V to 5 V of PSSH is shown. Similar I-V shape with Fig. 4b was observed in this PSSH system. Hence, we deduce that the observed switchable diode phenomenon arises from the PSSH phase separated regions inside the film of PEDOT PSS. However, the unipolar I-V sweep from 0 V up to −15 V of PSSH pristine film did not display hysteresis as shown in Fig. S4b; this fact suggests that the WORM phenomenon observed in Fig. 1a is not related solely to the PSSH regions.

The switchable diode effect in Fig. 2a,b cannot be explained by voltage modulating injection barrier discussed in various publications^18^,^19^ because the diode forward direction is antiparallel with applied voltage direction. We propose the following mechanism based on hole trapping and cation movements as discussed by Xie *et al.*^29^. The trapping of holes are possibly induced by redox reaction (electrolysis) of the adsorbed water in the film since PEDOT PSS is a hygroscopic material. The non-crossing to axis origin of IV curve after voltage sweeping from −5 V to 5 V shown in Fig. 2a inset indicated the presence of cation movements. When −5 V poling voltage is applied on top electrode, holes injected from bottom electrode will be trapped at the interface and protons H^+^ will be transported to cathode. The charges are trapped and protons are transported via the water molecules within the PEDOT PSS film through Grotthus mechanism where protons will be transported from one water molecule to another water molecule through Hydrogen bonding^30^. It is noteworthy that electrolysis of water in the film^31^ and the acidic PSS H in PEDOT PSS film are the possible sources of protons. At small voltage sweep from −1.5 V to 0 V (applied to top electrode), trapped holes hinder further injection which results in reverse- bias-like I-V in Fig. 2b. When voltage is swept from 0 V to 1.5 V, holes will be injected from anode and trapped holes are extracted at the cathode; this results in forward-bias- like I-V in Fig. 2b. The above discussed charge transport events occurred in the PSSH phase separation regions of PEDOT PSS film as revealed by control experiment Fig. S3a.

The reason for the moderate retention in Fig. 3b is possibly due to relaxation of trapped holes. Similarly, relaxation of accumulated oxygen vacancy was also proposed as the possible reason for short retention time in switchable diode in WO_3_^19^. A similar scenario was also employed to explain symmetrical NDR and switchable diode in TiO_2_ as discussed by Du *et al.*^32^. We also note similar I-V characteristic observed in water- redox based memory device of metal organic framework (MOF)^33^ and hydrated/Nickel decorated DNA^34^. However, in those systems (MOF and DNA), coexistence with WORM memory can hardly be achieved. Therefore, the coexistence of switchable diode and WORM memory in PEDOT PSS is a unique culmination of this class of memory material.

## Conclusion

In this work, we study the memory phenomena in PEDOT PSS. In the same film, there are two memory effects coexisted, namely the switchable diode effect and write once read many times memory. Originally, the pristine state of the device is in its high conducting state with Ohmic behaviour. After reduction voltage of −5 V, the junction switches to OFF state with low conduction and pristine state cannot be set back to high conducting state even after applying high positive or negative voltage. The drop in conduction is due to redox reaction that reduces PEDOT^+^ to PEDOT^0^ in the bulk film. After the film was reduced, the switchable diode arose. The switchable diode was explained due to the trapping of holes and proton transport in PEDOT PSS film under redox reaction of adsorbed water in the film. The switchable diode effect was originated from PSS H phase separated regions in the PEDOT PSS film.

The presented results broaden and deepen the understanding of memory phenomena in PEDOT PSS and extend further its applications in electronics and ionics devices including WORM nonvolatile memory, ionic diode and synapsis activity^11^,^35^,^36^,^37^.

## Methods

PEDOT PSS with 0.5 wt% PEDOT and 0.8 wt% PSS was purchased from Sigma Aldrich under code name 483095 Aldrich. The dark blue solution was filtered through 0.5 μm PTFE membrane to produce more homogenous solution; after that it was dilated in HCl and Deionized water for 1 day to further purify. The 6 μl solution was then drop-casted on gold coated Si substrate with 2 nm Ti adhesion layer to achieve a maximum 5 μm thick film with dark blue color; the standard deviation of measured thickness is about 1.71 μm. The film thickness was determined by surface profiler meter. The film was dried in air at about 60% humidity without heating. The Poly(4-styrenesulfonic acid) film (thickness of 60 μm) in control experiment was casted on Au substrate from 18 wt% in water solution purchased from Sigma Alrich under code name 561223 Alrich. The Deionized (DI) water in control experiment was obtained from Millipore system.

We conducted transport measurement using Keithley 4200 semiconductor analyser using bended gold metal probes with thickness of 300 μm and curvature arc of 40 μm pressing on the PEDOT PSS film as top electrode to study the physics of the memory switching phenomena. This avoids the interference of sputtered electrodes on resistive switching of the film. The contact area was 0.00005 cm^2^. We note that such strategy was also employed to study resistive switching in metal-organic framework^38^.

The film was characterized using micro Raman Spectroscopy with excitation laser wavelength of 488 nm, before and after electrical biasing. Optical image of pristine region and −5 V biased region was collected using Olympus optical microscopy. All of the experiments were performed at room temperature condition and 60% humidity.

## Additional Information

**How to cite this article**: Nguyen, V. C. and Lee, P. S. Resistive Switching Memory Phenomena in PEDOT PSS: Coexistence of Switchable Diode Effect and Write Once Read Many Memory. *Sci. Rep.* **6**, 19594; doi: 10.1038/srep19594 (2016).

## Supplementary Material

Supplementary Information
