# Supplementary material for: Resistive Switching Memory Phenomena in PEDOT PSS: Coexistence of Switchable Diode Effect and Write Once Read Many Memory
Source: Sci Rep. 2016 Jan 25;6:19594. doi: 10.1038/srep19594 (PMC4726348; doi:10.1038/srep19594)
Supplement: Supplementary Information [file srep19594-s1.doc]

Supplementary information

**Resistive Switching Memory Phenomena in PEDOT PSS: Coexistence of Switchable Diode Effect and Write Once Read Many Memory**

Viet Cuong Nguyen and Pooi See Lee*

*School of Materials Science and Engineering, Nanyang Technological University, 50 Nanyang Avenue, Singapore 639798, Singapore.*

*Email:* [*pslee@ntu.edu.sg*](mailto:pslee@ntu.edu.sg)

Fig S1 shows write once read many memory (WORM) states retention after storing the film in air for 3months. There is no significant degradation of current level. This fact suggests that PEDOT PSS is the air stable material and is in accordance with recent work of Bhansali et al [22]. Lack of degradation also implies that oxygen is not the main charge trapping center.

Fig S2a shows optical image of pristine area (lighter color) and area after biasing with -5V (dark color). Raman spectrum of the two area is shown in Fig S2b. Disappearing of Raman peak at 1267 cm^-1^ for -5V biasing indicates the film is reduced.

In Fig S3, effect of voltage sweep speed on current density peak at -2V of Fig 1b is shown. As the sweep rate is increased to 1V/s, the current density peak level at -2V will increase which suggests electrochemical reactions play important role.

In Fig S4 bipolar I-V sweep of pristine PSSH film on Au substrate was shown; the switchable diode effect was revealed in I-V hysteresis loop. Unipolar sweep from 0V to -15 V of pristine PSSH film shows almost no I-V hysteresis which indicates that write once read many memory does not come from PSSH .

In Fig S5, On state current and Off state current distribution of 20 tested devices from different batches are shown. The memory states can be distinguished clearly even with devices from different batches.

**Fig S1| WORM states retention after storing the device in air for 3months.**


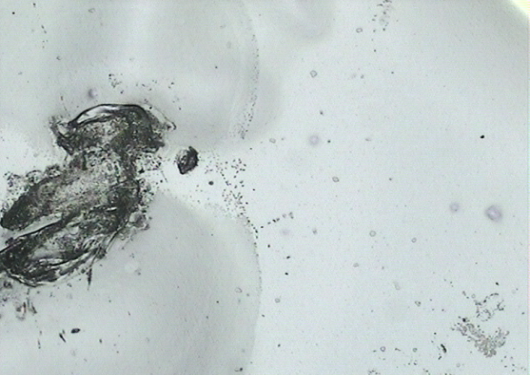


-5V Biased area

Pristine area

40 µm

1. (b)

**Fig S2| (a) Optical image of pristine area and area after being bias -5V (b) corresponding Raman spectra of each area.**

**Fig S3| Effect of voltage sweep speed on current density peak at -2V.**

1. (b)

**Fig S4| a) bipolar I-V sweeping of pristine PSSH film (b) unipolar I-V sweep of pristine PSSH film.**

**Fig S5| On state current and Off state current distribution of 20 tested devices from different batches.**
